# Association of C‐Reactive Protein‐Triglyceride Glucose Index With Chronic Obstructive Pulmonary Disease: Results From the NHANES and CHARLS Cohorts

**DOI:** 10.1155/mi/9592487

**Published:** 2026-07-04

**Authors:** Jianing Gan, Liuhan Chen, Yi Zhu

**Affiliations:** ^1^ The Fifth Affiliated Hospital of Zhengzhou University, Zhengzhou, 450000, Henan, China, ztzy.com; ^2^ Shanghai University of Sport, Shanghai, 200438, China, sus.edu.cn

**Keywords:** CHARLS, chronic obstructive pulmonary disease, C-reactive protein-triglyceride glucose index, inflammation, insulin resistance, NHANES

## Abstract

**Background:**

The C‐reactive protein (CRP)‐triglyceride glucose index (CTI) is a new composite biomarker used to assess inflammation and insulin resistance (IR) severity. Inflammation and IR play important roles in chronic obstructive pulmonary disease (COPD). However, the impact of CTI on COPD remains unknown.

**Methods:**

To explore the association between CTI and the prevalence of COPD, a total of 8682 participants from the China Health and Retirement Longitudinal Study (CHARLS) and 8986 participants from the US National Health and Nutrition Examination Survey (NHANES) were included. We used logistic multivariate regression to evaluate the association between CTI and COPD. In addition, smooth curve fitting analyzed dose–response relationships, while subgroup analyses explored effect heterogeneity.

**Results:**

We found a positive correlation between CTI and the risk of COPD after adjusting for all covariates in the NHANES database (OR = 1.34, 95% confidence interval [CI]: 1.18–1.53), which is consistent with the findings obtained from Cox regression analysis in the CHARLS database (HR = 1.16, 95% CI: 1.07–1.26), with consistent dose–response trends confirmed by restricted cubic spline (RCS) analyses. Subgroup analyses confirmed consistency across most strata, with a significant interaction detected with cardiovascular disease (CVD). Sensitivity analyses further confirmed the robustness of associations.

**Conclusions:**

These findings indicate a significant association between CTI and COPD, suggesting its potential role as a biomarker for the prevention and treatment of COPD.

## 1. Introduction

Chronic obstructive pulmonary disease (COPD) is a heterogeneous lung condition characterized by chronic respiratory symptoms (dyspnea, cough, sputum production, and/or exacerbations) due to abnormalities of the airways (bronchitis and bronchiolitis) and/or alveoli (emphysema) that cause persistent, often progressive, airflow obstruction [1]. In 2021, there were ~213.3 million prevalent cases of COPD worldwide, resulting in about 3.719 million deaths and 79.8 million disability‐adjusted life years [2]. While imposing a substantial health burden, the economic burden of COPD is expected to rise significantly in the coming decades [3]. Hence, early and accurate identification of COPD is crucial for its timely prevention and management [4]. Currently, spirometry serves as the “gold standard” for diagnosing COPD and assessing its severity, progression, prognosis, and treatment response [5]. However, in primary care settings, the utilization of pulmonary function tests is limited by factors such as a shortage of trained healthcare professionals and insufficient time allocation. Consequently, there is a pressing need to identify simple and effective biomarkers for COPD screening.

Diabetes mellitus and COPD frequently coexist, with studies indicating that ~10% of patients with type 2 diabetes mellitus (T2DM) also suffer from COPD [6]. The potential pathophysiological mechanisms involve multiple aspects: hyperglycemic conditions can enhance airway smooth muscle responsiveness to contractile stimuli while activating nonenzymatic glycation of pulmonary collagen and elastin, ultimately leading to impaired lung elasticity [7]. Imaging evidence demonstrates that insulin resistance (IR) induces airway wall thickening, thereby promoting airway inflammation and subsequent lung tissue damage [8]. As a surrogate marker for evaluating IR, the triglyceride‐glucose (TyG) index has been proven to be significantly associated with an increased risk of COPD development [9, 10].

The pathogenesis of COPD involves the activation of multiple inflammatory cells [11]. C‐reactive protein (CRP) is a widely recognized inflammatory biomarker and plays a crucial role in assessing infections and various inflammatory diseases [12]. Studies have shown that circulating CRP levels in patients with stable COPD are significantly higher than those in healthy controls, confirming the validity of CRP as a biomarker of COPD [13]. Multiple meta‐analyses indicate that elevated CRP concentrations are often observed in severe cases, reflecting aggravated inflammation and a progressive disease state [14, 15].

Ruan and colleagues proposed a novel composite index, the CRP‐triglyceride glucose index (CTI). This index integrates assessments of both inflammation and IR, thereby addressing a key limitation of the TyG index and CRP, which are confined to evaluating a single dimension [16]. The CTI has demonstrated significant value in clinical research, and the association of CTI with stroke, cancer, and cardiovascular disease (CVD) exposure effects has been validated by multiple studies [17–20]. However, its relationship with COPD remains lacking in clinical evidence. We hypothesized that higher CTI levels are associated with an increased risk of COPD.

Therefore, utilizing data from two large‐scale databases—the China Health and Retirement Longitudinal Study (CHARLS) and the National Health and Nutrition Examination Survey (NHANES)—this study aims to systematically investigate the association between CTI and COPD. The findings are expected to provide new epidemiological evidence for the early screening of COPD and the development of metabolic‐inflammatory coordinated intervention strategies.

## 2. Methods

### 2.1. Study Population

**Figure 1:**
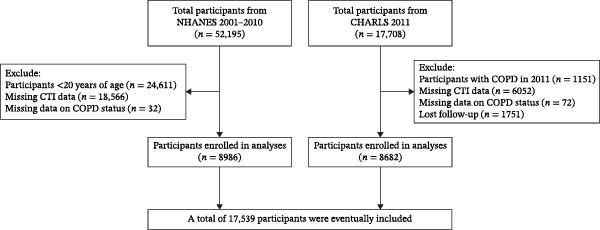
Flow chart of the study design.

This study is based on data from the NHANES and the CHARLS, two independent national cohorts. The NHANES, a nationally representative cross‐sectional survey utilizing complex, multistage, probability sampling methods, is designed and conducted by the National Center for Health Statistics (NCHS). This study analyzed five cycles of NHANES data from 2001 to 2010 for analysis, comprising 52,195 participants. We excluded individuals under 20 years of age, missing CTI data, and missing COPD data. Ultimately, a total of 8986 participants were eligible for further analysis (Figure 1). The NHANES has been approved by the NCHS Research Ethics Review Board and has obtained informed consent from all participants. CHARLS is a national longitudinal survey of Chinese adults aged 45 and older. This study used data from the 2011 baseline (Wave 1), including 17,708 participants. We excluded individuals with COPD at baseline, missing CTI data, and missing COPD data. After exclusions, 8682 participants were included to assess the relationship between CTI and COPD (Figure 1). This project was approved by the Institutional Review Board at Peking University Medical School, and all participants provided written informed consent (IRB00001052‐11014 and IRB00001052‐11015).

### 2.2. Calculation of CTI

The TyG index was computed using the following formula: TyG = ln[(TG (mg/dL) × fasting glucose (mg/dL))/2]. CRP and TyG, respectively, reflect the inflammatory and IR status of the subject and combine to form the CTI. The CTI index was calculated as follows: CTI = 0.412 × ln(CRP) + TyG.

### 2.3. Ascertainment of COPD

In the NHANES database, participants who answered “yes” to either the questions “Has a doctor ever told you had emphysema?” or “Has a doctor ever told you had chronic bronchitis?” in the questionnaire about medical conditions were classified as having COPD. In the CHARLS database, COPD was defined based on an affirmative answer to the question, “Has a doctor ever diagnosed you with COPD?”

### 2.4. Covariates

In the NHANES cohort, potential covariates included age, gender, race, education level (<High school, High school, >High school), marital status, family poverty income ratio (PIR) (≤1, 1.31–3.5, >3.5), body mass index (BMI) (<18.5, 18.5–23.9, 24−27.9, ≥28), smoking status, drinking status (never drinkers, moderate drinking defined as 1–2 drinks per day for men and 1 drink per day for women, heavy drinking defined as ≥3 drinks per day for men and ≥2 drinks per day for women), and self‐reported history of diabetes, hypertension, and CVD. In the CHARLS cohort, covariates included age, gender, education level, marital status, BMI, smoking status, drinking status (Never drinker, Former drinker, Current drinker), as well as disease information for diabetes, hypertension, and CVD.

In both studies, smoking status was divided into three groups: never (smoking <100 cigarettes), ever smoked (smoking ≥100 cigarettes but has quit), and currently smoking (smoking ≥100 cigarettes and consistently). Hypertension diagnosis criteria encompassed the average systolic blood pressure/average diastolic blood pressure ≥140/90 mmHg, taking antihypertensive drugs, and history of hypertension. Participants were diagnosed with diabetes if one of the following criteria was met: previous diagnosis of diabetes, taking diabetic pills, fasting glucose ≥7 mmol/L, or HbA1c ≥6.5%. If a participant answers “yes” to any of the five questions asking about their history of congestive heart failure, coronary heart disease, angina/angina pectoris, heart attack, and stroke, the participant is are considered to have CVD.

### 2.5. Statistical Analysis

We applied random forest imputation to missing covariates using R software (version 3.5.0) with the “missRanger” package. For the NHANES data, the complex survey design factors for the NHANES were considered, including sample weights, clustering, and stratification, to ensure national representative estimates. In the baseline characteristics table, weighted variables were estimated with normally distributed continuous variables reported as the mean ± standard deviation (SD), skewed distributional data reported as median (Quartile 1 and Quartile 3), and categorical variables reported as unweighted frequencies with weighted percentages. In the CHARLS cohort, unweighted analyses were performed. Continuous variables are presented as mean ± SD or median (Quartile 1 and Quartile 3), and categorical variables as frequencies with percentages. For continuous variables, intergroup comparisons were performed using the independent *t*‐test for normally distributed data and the Wilcoxon rank‐sum test for nonnormally distributed data. Categorical variables were compared between groups using the *χ* ^2^ test.

CTI was categorized into quartiles (Q1–Q4), with Q1 serving as the reference. To investigate the association between CTI and COPD prevalence, we applied weighted logistic regression analysis and Cox regression analysis and constructed three models to eliminate potential confounding effects: Model 1 (unadjusted), Model 2 (adjusted for age, gender, education, marital status, with an additional adjustment for race and PIR in the NHANES cohort), and Model 3 (further adjusted for BMI, smoking status, drinking status, diabetes, hypertension, and CVD). The Schoenfeld residual method was used to confirm the proportional‐hazard assumption, and no violations were detected (Supporting Information 1: Table S1). Variance inflation factors (VIFs) were calculated to assess multicollinearity. To characterize potential linear or nonlinear dose–response patterns between CTI and COPD, restricted cubic spline (RCS) curves were generated. We also stratified the logistic regression analyses by gender, smoking status, BMI, hypertension, and CVD. Receiver operating characteristic (ROC) curves and area under the curve (AUC) were utilized to assess the predictive capability of CTI, CRP, and the TyG index on the risk of COPD. DeLong’s method was used to calculate the 95% confidence intervals (CIs) for the AUC values and to perform pairwise comparisons of the AUC values between CTI and each individual marker. Additionally, to investigate the relationship between CTI and pulmonary function, we used multivariable linear regression models to analyze the associations of CTI with forced expiratory volume in 1 s (FEV1), forced vital capacity (FVC), and FEV1/FVC ratio in the NHANES (2007–2010 subsample), adjusting for all covariates. Furthermore, we performed multiple sensitivity analyses to test the robustness of the results. First, in order to further validate the robustness of the research results, we implemented multiple imputation, followed by conducting correlation analysis. Second, we repeated the correlation analysis after excluding subjects with extreme BMI values (<15 kg/m^2^ or >60 kg/m^2^). Third, we conducted an unweighted logistic regression analysis on the NHANES data. Finally, to avoid reverse causality, we excluded participants who developed COPD during the first follow‐up period in the CHARLS database from the analysis. All analyses were conducted using R statistical software (version 3.5.0). A two‐tailed *p*  < 0.05 was considered statistically significant.

## 3. Results

### 3.1. Baseline Characteristics

Among the 8986 participants in the NHANES cohort, 653 (7.27%) were considered to have COPD (Table 1). The mean age was 46.40 ± 16.76 years, and 48.36% of the participants were male. Individuals with COPD tended to be female, older, non‐Hispanic White, and have lower education levels and lower PIR. They were also more likely to be current or former smokers and heavy drinkers, with higher rates of obesity, hypertension, diabetes, and CVD. Furthermore, elevated levels of cholesterol, blood glucose, triglycerides, CRP, TyG, HbA1c, and CTI values were observed in COPD patients.

**Table 1:** Weighted characteristics of participants from NHANES 2001–2010, by the COPD status.

Characteristics	Total (*n* = 8986)	Non‐COPD (*n* = 8333)	COPD (*n* = 653)	*p*‐Value
Age (years)	46.40 ± 16.76	45.84 ± 16.69	53.64 ± 15.99	<0.001 ^∗^
Gender	—	—	—	<0.001 ^∗^
Male	4319 (48.36)	4053 (49.26)	266 (36.63)	—
Female	4667 (51.64)	4280 (50.74)	387 (63.37)	—
Race	—	—	—	<0.001 ^∗^
Mexican American	1729 (8.19)	1681 (8.62)	48 (2.52)	—
Other Hispanic	690 (4.26)	647 (4.40)	43 (2.47)	—
Non‐Hispanic White	4465 (70.38)	4043 (69.68)	422 (79.55)	—
Non‐Hispanic Black	1698 (11.20)	1586 (11.30)	112 (9.91)	—
Other Race	404 (5.97)	376 (6.00)	28 (5.55)	—
Education level	—	—	—	<0.001 ^∗^
<High school	2567 (18.43)	2344 (17.93)	223 (24.89)	—
High school	2148 (24.71)	2008 (24.86)	140 (22.71)	—
>High school	4271 (56.87)	3981 (57.21)	290 (52.40)	—
Marital status	—	—	—	<0.001 ^∗^
Married/living with partner	5578 (65.05)	5217 (65.44)	361 (59.98)	—
Widowed/divorced/separated	1975 (17.92)	1756 (16.97)	219 (30.14)	—
Never married	1433 (17.04)	1360 (17.59)	73 (9.89)	—
PIR	—	—	—	<0.001 ^∗^
<1.3	2675 (19.92)	2436 (19.35)	239 (27.27)	—
1.3–3.5	3571 (37.71)	3308 (37.45)	263 (41.08)	—
≥3.5	2740 (42.37)	2589 (43.20)	151 (31.65)	—
BMI	—	—	—	<0.001 ^∗^
Underweight	127 (1.59)	101 (1.45)	26 (3.36)	—
Normal	2492 (30.72)	2350 (31.37)	142 (22.37)	—
Overweight	3103 (33.32)	2908 (33.56)	195 (30.18)	—
Obesity	3264 (34.37)	2974 (33.62)	290 (44.09)	—
Smoking status	—	—	—	<0.001 ^∗^
Never	4790 (52.70)	4585 (54.33)	205 (31.56)	—
Former	2325 (24.82)	2104 (24.31)	221 (31.34)	—
Current	1871 (22.48)	1644 (21.35)	227 (37.09)	—
Drinking status	—	—	—	<0.001 ^∗^
Never	1539 (13.62)	1443 (13.72)	96 (12.40)	—
Moderate	3229 (37.92)	3014 (38.39)	215 (31.86)	—
Heavy	4218 (48.46)	3876 (47.89)	342 (55.74)	—
Glucose (mg/dL)	98.00 (91.00, 106.00)	98.00 (91.00, 106.00)	100.00 (92.00, 111.00)	<0.001 ^∗^
Triglyceride (mg/dL)	111.00 (78.00, 162.00)	110.00 (77.00, 160.00)	129.00 (88.87, 184.25)	<0.001 ^∗^
HDL‐C (mg/dL)	52.00 (43.00, 63.00)	52.00 (43.00, 63.00)	50.00 (42.00, 64.00)	0.350
LDL‐C (mg/dL)	116.46 ± 34.97	116.35 ± 34.94	117.89 ± 35.34	0.370
Cholesterol (mg/dL)	193.00 (168.00, 222.00)	193.00 (168.00, 221.00)	198.00 (169.00, 229.00)	0.015 ^∗^
CRP (mg/dL)	0.18 (0.07, 0.44)	0.18 (0.07, 0.43)	0.30 (0.12, 0.71)	<0.001 ^∗^
TyG index	8.61 (8.22, 9.05)	8.60 (8.21, 9.03)	8.80 (8.37, 9.20)	<0.001 ^∗^
HbA1c (%)	5.40 (5.10, 5.70)	5.40 (5.10, 5.70)	5.50 (5.20, 5.90)	<0.001 ^∗^
Hypertension	—	—	—	<0.001 ^∗^
No	5295 (64.49)	5022 (65.68)	273 (49.07)	—
Yes	3691 (35.51)	3311 (34.32)	380 (50.93)	—
Diabetes	—	—	—	<0.001 ^∗^
No	7555 (88.38)	7051 (88.81)	504 (82.92)	—
Yes	1431 (11.62)	1282 (11.19)	149 (17.08)	—
CVD	—	—	—	<0.001 ^∗^
No	8189 (93.15)	7692 (94.14)	497 (80.25)	—
Yes	797 (6.85)	641 (5.86)	156 (19.75)	—
CTI	7.96 ± 0.93	7.93 ± 0.92	8.33 ± 0.96	<0.001 ^∗^
CTI group	—	—	—	<0.001 ^∗^
Q1	2247 (28.60)	2156 (29.60)	91 (15.65)	—
Q2	2247 (25.45)	2089 (25.50)	158 (24.89)	—
Q3	2246 (23.69)	2083 (23.72)	163 (23.20)	—
Q4	2246 (22.26)	2005 (21.18)	241 (36.27)	—

*Note:* Data are presented as *N* (%) (*χ* ^2^ test), median (P25, P75) (Wilcoxon test), and mean ± SD (independent *t*‐test). PIR, family poverty income ratio.

Abbreviations: BMI, body mass index; CRP, C‐reactive protein; CTI, C‐reactive protein‐triglyceride glucose index; CVD, cardiovascular disease; HDL‐C, high‐density lipoprotein cholesterol; LDL‐C, low‐density lipoprotein cholesterol; TyG, triglyceride‐glucose.

^∗^ *p*  < 0.05.

Among the 8682 participants in the CHARLS cohort, the median age was 50 years, with females comprising 55.69% (Table 2). Individuals with COPD were also typically male, older, with lower education levels, underweight, with a history of smoking or alcohol consumption, and face a higher risk of CVD and hypertension. Moreover, patients with COPD exhibited elevated CRP, HbA1c, and CTI levels.

**Table 2:** Characteristics of participants from CHARLS 2011–2018, by the COPD status.

Characteristics	Total (*n* = 8682)	Non‐COPD (*n* = 7597)	COPD (*n* = 1085)	*p*‐Value
Age (years)	50 (57, 63)	50 (56, 63)	53 (59, 66)	<0.001 ^∗^
Gender	—	—	—	<0.001 ^∗^
Male	3847 (44.31)	3295 (43.37)	552 (50.88)	—
Female	4835 (55.69)	4302 (56.6)	533 (49.12)	—
Education level	—	—	—	0.008 ^∗^
<High school	7799 (89.83)	6797 (89.47)	1002 (92.35)	—
High school	784 (9.03)	707 (9.31)	77 (7.10)	—
>High school	99 (1.14)	93 (1.22)	6 (0.55)	—
Marital status	—	—	—	<0.001 ^∗^
Married/living with partner	7793 (89.76)	6855 (90.23)	938 (86.45)	—
Widowed/divorced/separated	833 (9.59)	696 (9.16)	137 (12.63)	—
Never married	56 (0.65)	46 (0.61)	10 (0.92)	—
BMI	—	—	—	<0.001 ^∗^
Underweight	497 (5.72)	396 (5.21)	101 (9.31)	—
Normal	3616 (41.65)	3163 (41.63)	453 (41.75)	—
Overweight	1802 (20.76)	1593 (20.97)	209 (19.26)	—
Obesity	2767 (31.87)	2445 (32.18)	322 (29.68)	—
Smoking status	—	—	—	<0.001 ^∗^
Never	5546 (63.88)	4954 (65.21)	592 (54.56)	—
Former	643 (7.41)	524 (6.90)	119 (10.97)	—
Current	2493 (28.71)	2119 (27.89)	374 (34.47)	—
Drinking status	—	—	—	<0.001 ^∗^
Never	5412 (62.34)	4789 (63.04)	623 (57.42)	—
Former	685 (7.89)	556 (7.32)	129 (11.89)	—
Current	2585 (29.77)	2252 (29.64)	333 (30.69)	—
Glucose (mg/dL)	94.32 (102.42, 113.09)	94.50 (102.42, 113.22)	93.96 (102.06, 112.68)	0.347
Triglyceride (mg/dL)	76.11 (107.08, 156.65)	76.11 (106.20, 155.76)	75.22 (107.97, 160.18)	0.492
HDL‐C (mg/dL)	39.82 (49.10, 59.54)	39.82 (48.71, 59.15)	39.82 (49.87, 60.12)	0.333
LDL‐C (mg/dL)	93.17 (114.43, 136.86)	93.56 (114.82, 137.24)	92.78 (112.50, 135.70)	0.197
Cholesterol (mg/dL)	166.62 (190.21, 214.95)	167.01 (190.21, 214.95)	166.43 (190.98, 215.53)	0.795
CRP (mg/dL)	0.53 (0.99, 2.03)	0.52 (0.97, 1.97)	0.60 (1.09, 2.50)	<0.001 ^∗^
TyG index	8.23 (8.61, 9.07)	8.23 (8.61, 9.07)	8.25 (8.60, 9.08)	0.685
HbA1c (%)	4.90 (5.10, 5.40)	4.90 (5.10, 5.40)	4.90 (5.20, 5.50)	0.005 ^∗^
Hypertension	—	—	—	0.028 ^∗^
No	5305 (61.10)	4675 (61.54)	630 (58.06)	—
Yes	3377 (38.90)	2922 (38.46)	455 (41.94)	—
Diabetes	—	—	—	0.678
No	7276 (83.81)	6362 (83.74)	914 (84.24)	—
Yes	1406 (16.19)	1235 (16.26)	171 (15.76)	—
CVD	—	—	—	<0.001 ^∗^
No	7720 (88.92)	6833 (89.94)	887 (81.75)	—
Yes	962 (11.08)	764 (10.06)	198 (18.25)	—
CTI	8.14 (8.66, 9.25)	8.14 (8.65, 9.23)	8.18 (8.73, 9.36)	0.002 ^∗^
CTI group	—	—	—	0.011 ^∗^
Q1	2184 (25.16)	1927 (25.37)	257 (23.69)	—
Q2	2168 (24.97)	1928 (25.38)	240 (22.12)	—
Q3	2182 (25.13)	1899 (25.00)	283 (26.08)	—
Q4	2148 (24.74)	1843 (24.26)	305 (28.11)	—

*Note:* Data are presented as *N* (%) (χ^2^ test), median (P25, P75) (Wilcoxon test) and mean ± SD (independent *t*‐test).

Abbreviations: BMI, body mass index; CRP, C‐reactive protein; CTI, C‐reactive protein‐triglyceride glucose index; CVD, cardiovascular disease; HDL‐C, high‐density lipoprotein cholesterol; LDL‐C, low‐density lipoprotein cholesterol; TyG, triglyceride‐glucose.

^∗^ *p*  < 0.05.

### 3.2. Cross‐Sectional Correlation Between CTI and COPD

The primary objective of this study was to explore the association between CTI and COPD. Prior to conducting regression analyses, we evaluated multicollinearity by calculating the VIF (Supporting Information 2: Table S2). All VIF values were below 5, indicating no significant multicollinearity among the variables. We adopted weighted multivariable logistic regression analysis using three models, with the results presented in Table 3. After adjusting for confounders, all three models demonstrated a significant positive association between CTI and COPD (*p*  < 0.001). When CTI was treated as a categorical variable, compared with Q1, progressively higher quartiles showed an increase in COPD risk (*p* for trend = 0.002): Q2 (OR 1.49, 95% CI: 1.04−2.14), Q3 (OR 1.26, 95% CI: 0.86–1.85), and Q4 (OR 1.97, 95% CI: 1.41–2.75). Additionally, CTI was negatively associated with the FEV1/FVC ratio in multivariable linear regression analysis (*β* = −0.004, 95% CI: −0.006, −0.001, *p* = 0.007) (Supporting Information 3: Table S3).

**Table 3:** Association between CTI and the risk of COPD from NHANES.

Characteristic	Model 1		Model 2		Model 3	
	OR (95% CI)	*p* value	OR (95% CI)	*p* value	OR (95% CI)	*p* value
CTI	1.57 (1.43–1.73)	<0.001 ^∗^	1.46 (1.31–1.61)	<0.001 ^∗^	1.34 (1.18–1.53)	<0.001 ^∗^
CTI group						
Q1	Ref.		Ref.		Ref.	
Q2	1.85 (1.34–2.54)	<0.001 ^∗^	1.66 (1.18–2.34)	0.004 ^∗^	1.49 (1.04–2.14)	0.031 ^∗^
Q3	1.85 (1.33–2.58)	<0.001 ^∗^	1.54 (1.09–2.18)	0.016 ^∗^	1.26 (0.86–1.85)	0.226
Q4	3.24 (2.45–4.28)	<0.001 ^∗^	2.60 (1.96–3.45)	<0.001 ^∗^	1.97 (1.41–2.75)	<0.001 ^∗^
*p* for trend	<0.001 ^∗^		<0.001 ^∗^		0.002 ^∗^	

*Note:* Model 1 = Crude. Model 2 = age, gender, race, education level, marital status, and PIR were adjusted. Model 3 = Model 2 + smoking status, drinking status, BMI, diabetes, hypertension, and CVD were adjusted.

Abbreviations: CI, confidence interval; OR, odds ratio.

^∗^ *p*  < 0.05.

### 3.3. Longitudinal Association Between CTI and COPD Risk

During an average follow‐up period of 8 years in CHARLS, 1085 (12.50%) participants experienced their first COPD. The results of Cox regression analysis are presented in Table 4. After adjusting for covariates, each one‐unit increase in CTI was associated with a 16% higher risk of COPD (HR: 1.16, 95% CI: 1.07–1.26, *p*  < 0.001). Furthermore, compared to Q1, the risk of COPD increased with rising CTI levels (*p* for trend = 0.001), with a significantly elevated risk observed in Q4 (HR 1.31, 95% CI: 1.10–1.58).

**Table 4:** Longitudinal association between CTI and the risk of COPD from CHARLS.

Characteristic	Model 1		Model 2		Model 3	
	HR (95% CI)	*p* value	HR (95% CI)	*p* value	HR (95% CI)	*p* value
CTI	1.11 (1.04–1.19)	0.002 ^∗^	1.11 (1.04–1.19)	0.003 ^∗^	1.16 (1.07–1.26)	<0.001 ^∗^
CTI group						
Q1	Ref.		Ref.		Ref.	
Q2	0.94 (0.79–1.12)	0.468	0.91 (0.76–1.08)	0.283	0.93 (0.78–1.11)	0.422
Q3	1.11 (0.93–1.31)	0.242	1.09 (0.92–1.29)	0.326	1.13 (0.95–1.35)	0.615
Q4	1.23 (1.04–1.45)	0.016 ^∗^	1.21 (1.02–1.43)	0.026 ^∗^	1.31 (1.10–1.58)	0.003 ^∗^
*p* for trend	0.004 ^∗^		0.005 ^∗^		0.001 ^∗^	

*Note:* Model 1 = Crude. Model 2 = age, gender, education level, and marital status were adjusted. Model 3 = Model 2 + smoking status, drinking status, BMI, diabetes, hypertension, and CVD were adjusted.

Abbreviations: CI, confidence interval; HR, hazard ratio.

^∗^ *p*  < 0.05.

### 3.4. RCS Analysis Investigating the Relationship Between CTI and COPD

**Figure 2:**
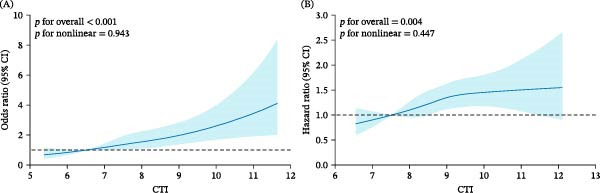
Restricted cubic spline plots of the association between CTI and COPD. (A) A dose–response curve from the NHANES cohort. (B) A dose–response curve from the CHARLS cohort.

To evaluate the dose–response relationship between CTI and COPD, we performed RCS regression analysis (Figure 2). After adjusting for covariates, a significant positive linear association between CTI and COPD was observed in both the NHANES and CHARLS cohorts (NHANES: nonlinear *p*‐value = 0.943 and CHARLS: nonlinear *p*‐value = 0.447).

### 3.5. Subgroup Analysis

**Figure 3:**
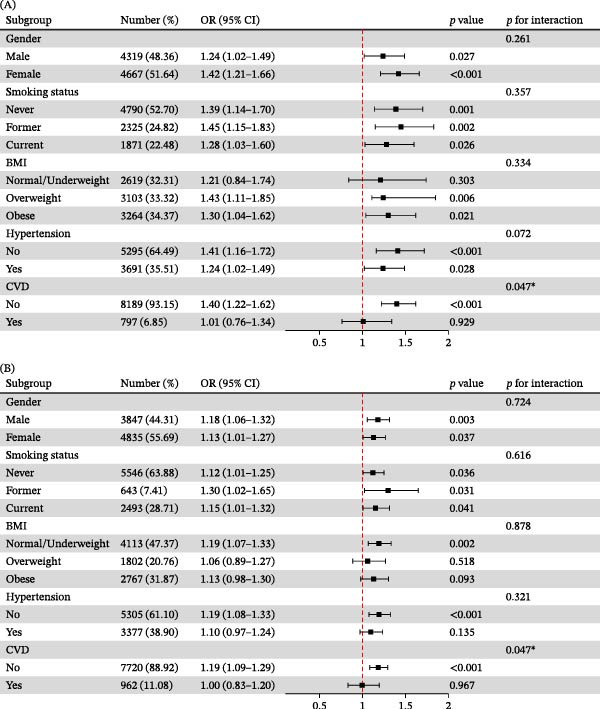
The relationship between CTI and the risk of COPD according to different subgroups. (A) A forest plot from the NHANES cohort. (B) A forest plot from the CHARLS cohort,  ^∗^ *p* < 0.05.

To explore differences in the relationship between CTI and COPD across different populations, we performed subgroup analyses stratified by gender, smoking status, BMI, hypertension, and CVD for both the CHARLS and NHANES cohorts (Figure 3). In the NHANES cohort, the association between CTI and COPD remained consistent across subgroups stratified by gender, smoking status, and hypertension. A significant correlation between CTI and COPD was observed only in overweight/obese individuals and non‐CVD patients, with a significant interaction detected between CTI and CVD (*p* for interaction = 0.047). In the CHARLS cohort, after stratification, the effect of CTI on COPD remained consistent across gender and smoking status subgroups. Additionally, a significant interaction was observed between CTI and CVD (*p* for interaction = 0.047).

### 3.6. The Predictive Capability of CTI, CRP, and TyG Index on COPD

**Figure 4:**
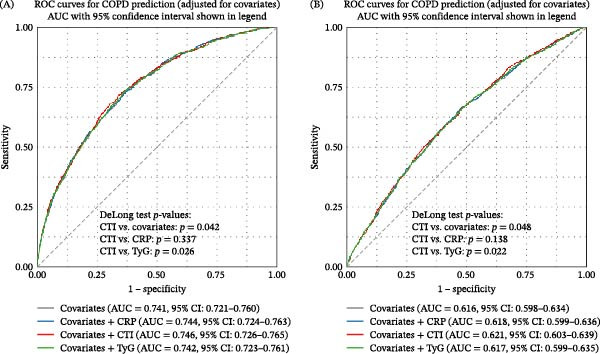
ROC curves for CRP, TyG index, and CTI predicting COPD incidence after adjustment for covariates. (A) ROC curves for CRP, TyG index, and CTI, adjusted for covariates in NHANES. (B) ROC curves for CRP, TyG index, and CTI, adjusted for covariates in CHARLS.

To compare the predictive value of CTI, CRP, and TyG for COPD risk, we performed ROC curve analyses (Figure 4). The ROC curves showed that CTI had the highest predictive ability for COPD occurrence in NHANES (AUC: 0.746, 95% CI: 0.726–0.765), followed by CRP (AUC: 0.744, 95% CI: 0.724–0.763) and the TyG index (AUC: 0.742, 95% CI: 0.723–0.761). Pairwise comparison using DeLong’s test revealed that the difference between CTI and TyG was statistically significant (*p* = 0.026), while no significant difference was observed between CTI and CRP (*p* = 0.337). Results from CHARLS were consistent with NHANES, with CTI demonstrating the highest predictive performance (AUC: 0.621, 95% CI: 0.603–0.639), followed by CRP (AUC: 0.618, 95% CI: 0.599–0.636), and then the TyG index (AUC: 0.617, 95% CI: 0.599–0.635). DeLong’s test revealed that the difference between CTI and TyG was statistically significant (*p* = 0.022), while no significant difference was observed between CTI and CRP (*p* = 0.138).

### 3.7. Sensitivity Analysis

To test the robustness of the results, we conducted sensitivity analyses. Removal of BMI outliers (<15 kg/m^2^) and (>60 kg/m^2^) did not significantly affect the results of correlation (Supporting Information 4: Table S4). In addition, after performing multiple imputations, similar results were still obtained (Supporting Information 5: Table S5). Furthermore, using unweighted logistic regression for the NHANES cohort, the association between CTI and COPD remained robust (Supporting Information 6: Table S6). To minimize reverse causality bias, we repeated the analysis after excluding individuals who were diagnosed with COPD during the first follow‐up period in the CHARLS cohort, and the results remained stable (Supporting Information 7: Table S7).

## 4. Discussion

To the best of our knowledge, this study, for the first time, integrated data from the CHARLS and NHANES databases to systematically investigate the associations between CTI and COPD. The study found a significant positive association between CTI and COPD, and we confirmed the robustness of our findings using various statistical approaches, highlighting the importance of CTI as a potential biomarker for COPD. Moreover, dose–response analyses demonstrated a linear relationship between CTI and COPD. Consistently, CTI was also negatively associated with the FEV1/FVC ratio, further supporting its potential as a biomarker reflecting airflow obstruction.

CTI is a composite biological indicator that integrates CRP and the TyG index, where CRP is used to assess the inflammatory status and the TyG index is used for IR. Several cross‐sectional studies utilizing the NHANES database have demonstrated a significant positive association between the TyG index and COPD [21, 22], which is consistent with our research findings. Furthermore, various TyG‐related obesity indices, such as TyG‐WHtR, TyG‐WC, and TyG‐BMI, have also been found to be positively associated with COPD [22]. Moreover, studies have shown that the TyG index serves as a marker for COPD risk specifically in women, further supporting its validity as a biomarker [10]. In addition, multiple studies have confirmed that CRP exhibits a significant positive correlation with the severity of COPD and patient mortality [23, 24]. He et al. [9] found that the combined elevation of TyG and CRP significantly increased the risk of COPD, which aligns with our findings. Furthermore, our study integrated TyG and CRP into a stable composite index. The ROC curve demonstrated that this composite index exhibited superior predictive ability for COPD compared to CRP or the TyG index alone, with a statistically significant difference between CTI and TyG. These results support the potential utility of the CTI index as an effective tool for identifying COPD risk.

Our findings indicate a positive correlation between the CTI index and COPD. Under IR conditions, the expression of TGF‐β1 in bronchial epithelial cells is significantly upregulated, promoting pulmonary fibrosis and airway remodeling, which leads to airway inflammation [25]. Moreover, IR disrupts glucose metabolism in airway epithelial cells, compromises barrier function, and heightens airway sensitivity to inflammatory insults. CRP is a well‐established systemic inflammatory biomarker. Studies have demonstrated that serum high‐sensitivity CRP levels are significantly elevated in patients with COPD, showing a negative correlation with FEV1 and 6‐min walk distance and maintaining an independent association with the GOLD stage [26]. CRP can activate oxidative stress pathways, upregulating transcription factors such as nuclear factor κB (NF‐κB) [27]. The resulting excess reactive oxygen species (ROS) oxidatively inactivate antiproteinases and damage lung tissue structure [28]. Furthermore, transcription factors of the NF‐κB family are known to activate the expression of numerous proinflammatory genes that are implicated in pulmonary inflammation [29].

Subgroup analysis further corroborated the robustness of the association between CTI and COPD. It is noteworthy that significant interactions between CTI and CVD were observed in two independent cohorts, where the association between CTI and COPD risk was significant only among individuals without CVD. CVD itself represents a strong chronic inflammatory state, characterized by pre‐existing high‐level systemic inflammatory responses. This condition may reach or exceed the additional inflammatory threshold that CTI is able to discern [30]. On the other hand, patients with CVD typically receive multiple pharmacological treatments, including statins, antiplatelet agents, angiotensin‐converting enzyme inhibitors (ACEI), and beta‐blockers [31]. These medications may attenuate the original association between CTI and COPD risk by modulating the components of CTI. Studies have shown that statins not only lower lipid levels but also possess significant anti‐inflammatory properties, potentially reducing CRP levels and improving insulin sensitivity [32]. Similarly, ACEI and beta‐blockers have been demonstrated to ameliorate metabolic profiles and mitigate inflammatory responses [33].

Our findings indicate that CTI is superior to CRP and the TyG index in predicting the risk of COPD. The underlying reason is that CTI can capture the synergistic pathophysiological mechanisms that these individual indicators overlook. Studies have found that proinflammatory cytokines increase the serine phosphorylation of the insulin receptor substrate (IRS), inhibiting its normal tyrosine phosphorylation, thereby blocking downstream insulin signaling and leading to IR [34]. The chronic stress state in COPD patients leads to abnormal activation of the hypothalamic–pituitary–adrenal (HPA) axis, resulting in elevated cortisol levels. Cortisol directly induces and exacerbates IR by promoting gluconeogenesis, inhibiting glucose utilization, and inducing protein catabolism [35]. Furthermore, adipose tissue in COPD patients exhibits a chronic inflammatory state, releasing excessive free fatty acids and proinflammatory factors, which further exacerbate IR through lipotoxicity and inflammatory responses [36]. In addition, the integrated advantages of CTI enable it to more comprehensively reflect the complex pathological mechanisms of COPD, encompassing multiple dimensions such as chronic inflammation, metabolic disorders, and tissue damage [37]. Therefore, CTI, as a simple, cost‐effective, and clinically accessible composite biomarker, demonstrates significant clinical application value in COPD risk prediction and early screening.

The present study has several strengths. First, this was the first study to investigate the association between CTI and the risk of COPD. Second, this study employed a composite index, CTI, which integrates the well‐validated CRP and the TyG index. This approach simultaneously captures deteriorating metabolic function and assesses the inflammatory status, thereby enabling a more comprehensive and sensitive evaluation of COPD risk. Third, our study comprehensively investigated the association between CTI and COPD using both continuous and categorical CTI variables in two nationally representative cohorts with distinct demographic characteristics. Nonetheless, there were several limitations to this study. First, while the longitudinal analysis offers some insight into the temporal relationship between CTI and COPD, further experimental studies are required to confirm causality. Second, although our analysis adjusted for multiple potential confounding factors, the possibility of residual confounding due to unmeasured or uncontrolled variables cannot be ruled out. Third, the samples were mainly from the United States and China, which limited the broad applicability of our findings to other demographic groups.

## 5. Conclusions

In summary, cohort findings from both countries indicated that CTI is independently positively associated with COPD. This study expands the evidence regarding the relationship between CTI and COPD and highlights the potential of CTI as a tool for early detection and intervention of COPD. Further studies are needed to confirm the causal relationship between CTI and COPD and to elucidate the underlying mechanisms.

## Funding

This work was supported by the National Natural Science Foundation of China Project (Grant 82274622).

## Conflicts of Interest

The authors declare no conflicts of interest.

## Supporting Information

Additional supporting information can be found online in the Supporting Information section.

## Supporting information


**Supporting Information 1** Table S1: Schoenfeld residual test for proportional hazards assumption.

**Supporting Information 2** Table S2: Results of Generalized Variance Inflation Factor (GVIF) Analysis.

**Supporting Information 3** Table S3: The association of CTI with pulmonary function after adjusting for relevant covariates in the NHANES cohort.

**Supporting Information 4** Table S4: Association between CTI and the risk of SI after excluding BMI < 15 and > 60 kg/m^2^.

**Supporting Information 5** Table S5: Association between CTI and the risk of COPD after multiple imputation.

**Supporting Information 6** Table S6: Association between CTI and the risk of COPD using unweighted logistic regression.

**Supporting Information 7** Table S7: Association between CTI and the risk of COPD excluding participants with COPD during the first follow‐up period.

## Data Availability

The datasets generated and analyzed during the current study are available on the CHARLS and NHANES websites, available at https://wwwn.cdc.gov/nchs/nhanes/Default.aspx and http://charls.pku.edu.cn/en, respectively.
