# Supplementary material for: Association of C‐Reactive Protein‐Triglyceride Glucose Index With Chronic Obstructive Pulmonary Disease: Results From the NHANES and CHARLS Cohorts
Source: Mediators Inflamm. 2026 Jul 4;2026:9592487. doi: 10.1155/mi/9592487 (PMC13332394; doi:10.1155/mi/9592487)
Supplement: Supplementary file 1 — Supporting Information 1 Table S1: Schoenfeld residual test for proportional hazards assumption. [file MI-2026-9592487-s001.docx]

**Table S1** Schoenfeld residual test for proportional hazards assumption.

|  | χ² | *P* Value |
| --- | --- | --- |
| CTI | 1.672 | 0.196 |
| Age | 3.237 | 0.072 |
| Gender | 0.782 | 0.377 |
| Education level | 1.156 | 0.282 |
| Marital status | 2.228 | 0.136 |
| BMI | 1.236 | 0.266 |
| Smoking staus | 0.560 | 0.454 |
| Drinking status | 0.790 | 0.374 |
| CVD | 1.182 | 0.277 |
| Diabetes | 0.033 | 0.856 |
| Hypertension | 0.068 | 0.794 |
| Global | 20.812 | 0.053 |

Abbreviations: CTI, C-reactive protein-triglyceride glucose index; BMI, body mass index; CVD, cardiovascular disease.

*P<0.05
