# Supplementary material for: Association of C‐Reactive Protein‐Triglyceride Glucose Index With Chronic Obstructive Pulmonary Disease: Results From the NHANES and CHARLS Cohorts
Source: Mediators Inflamm. 2026 Jul 4;2026:9592487. doi: 10.1155/mi/9592487 (PMC13332394; doi:10.1155/mi/9592487)
Supplement: Supplementary file 2 — Supporting Information 2 Table S2: Results of Generalized Variance Inflation Factor (GVIF) Analysis. [file MI-2026-9592487-s002.docx]

| TableS2 Results of Generalized Variance Inflation Factor (GVIF) Analysis | | | |
| --- | --- | --- | --- |
| NHANES | | CHARLS | |
| variables | GVIF | variables | GVIF |
| Age | 1.376 | Age | 1.108 |
| Gender | 1.077 | Gender | 1.495 |
| Education level | 1.076 | Education level | 1.011 |
| Marital status | 1.077 | Marital status | 1.036 |
| BMI | 1.052 | BMI | 1.051 |
| Smoking status | 1.117 | Smoking status | 1.191 |
| Drinking status | 1.097 | Drinking status | 1.099 |
| Hypertension | 1.176 | Hypertension | 1.073 |
| Diabetes | 1.129 | Diabetes | 1.093 |
| CVD | 1.092 | CVD | 1.026 |
| CTI | 1.181 | CTI | 1.146 |
| Race | 1.030 |  |  |
| PIR | 1.071 |  |  |
| BMI, body mass index; LDL-c, low-density lipoprotein cholesterol; HDL-c, high-density lipoprotein cholesterol; HbA1c, Hemoglobin A1c | | | |
