# Supplementary material for: Association of C‐Reactive Protein‐Triglyceride Glucose Index With Chronic Obstructive Pulmonary Disease: Results From the NHANES and CHARLS Cohorts
Source: Mediators Inflamm. 2026 Jul 4;2026:9592487. doi: 10.1155/mi/9592487 (PMC13332394; doi:10.1155/mi/9592487)
Supplement: Supplementary file 3 — Supporting Information 3 Table S3: The association of CTI with pulmonary function after adjusting for relevant covariates in the NHANES cohort. [file MI-2026-9592487-s003.docx]

**Table S3** The association of CTI with pulmonary function after adjusting for relevant covariates in the NHANES cohort.

| NHANES | FEV1 | | FVC | | FEV1/FVC | |
| --- | --- | --- | --- | --- | --- | --- |
|  | β(95%CI) | *P* Value | β(95%CI) | *P* Value | β(95%CI) | *P* Value |
| **CTI** | -0.014 (-0.036, 0.008) | 0.200 | 0.001 (-0.027, 0.028) | 0.974 | -0.004 (-0.006, -0.001) | 0.007* |
| **CTI Group** |  |  |  |  |  |  |
| Q1 | Ref. |  | Ref. |  | Ref. |  |
| Q2 | 0.035 (-0.015, 0.085) | 0.168 | 0.084 (0.022, 0.147) | 0.008* | -0.007 (-0.013, -0.001) | 0.026* |
| Q3 | -0.021 (-0.075, 0.033) | 0.444 | 0.014 (-0.054, 0.081) | 0690 | -0.007 (-0.013, -0.001) | 0.029* |
| Q4 | -0.054 (-0.114, 0.006) | 0.076 | -0.023 (-0.098, 0.053) | 0.552 | -0.008 (-0.015, -0.001) | 0.025* |

Multiple linear regression models were adjusted by age, gender, race, education level, marital status, PIR, smoking status, drinking status, BMI, diabetes, hypertension, and CVD. Abbreviations: FEV1, forced expiratory volume in the first 1.0 s; FVC, forced vital capacity; CI, confidence interval.

*P<0.05
