# Supplementary material for: Association of C‐Reactive Protein‐Triglyceride Glucose Index With Chronic Obstructive Pulmonary Disease: Results From the NHANES and CHARLS Cohorts
Source: Mediators Inflamm. 2026 Jul 4;2026:9592487. doi: 10.1155/mi/9592487 (PMC13332394; doi:10.1155/mi/9592487)
Supplement: Supplementary file 4 — Supporting Information 4 Table S4: Association between CTI and the risk of SI after excluding BMI < 15 and > 60 kg/m2. [file MI-2026-9592487-s004.docx]

**Table S4** Association between CTI and the risk of SI after excluding BMI<15kg/m^2^ and >60kg/m^2^.

| NHANES | Model 1 | | Model 2 | | Model 3 | |
| --- | --- | --- | --- | --- | --- | --- |
|  | OR(95%CI) | *P* Value | OR(95%CI) | *P* Value | OR(95%CI) | *P* Value |
| **CTI** | 1.57 (1.43-1.73) | <0.001* | 1.46 (1.32-1.62) | <0.001* | 1.34 (1.18-1.53) | <0.001* |
| **CTI Group** |  |  |  |  |  |  |
| Q1 | Ref. |  | Ref. |  | Ref. |  |
| Q2 | 1.87 (1.36-2.57) | <0.001* | 1.68 (1.19-2.36) | 0.004* | 1.50 (1.04-2.15) | 0.029* |
| Q3 | 1.86 (1.33-2.61) | <0.001* | 1.55 (1.08-2.20) | 0.017* | 1.26 (0.86-1.86) | 0.234 |
| Q4 | 3.26 (2.47-4.31) | <0.001* | 2.62 (1.97-3.48) | <0.001* | 1.97 (1.41-2.75) | <0.001* |
|  | | | | | | |
| CHARLS | Model 1 | | Model 2 | | Model 3 | |
|  | HR(95%CI) | *P* Value | HR(95%CI) | *P* Value | HR(95%CI) | *P* Value |
| **CTI** | 1.11 (1.04-1.19) | 0.002* | 1.11 (1.04-1.19) | 0.002* | 1.16 (1.07-1.26) | <0.001* |
| **CTI Group** |  |  |  |  |  |  |
| Q1 | Ref. |  | Ref. |  | Ref. |  |
| Q2 | 0.91 (0.76-1.09) | 0.306 | 0.88 (0.74-1.06) | 0.172 | 0.90 (0.76-1.08) | 0.261 |
| Q3 | 1.09 (0.92-1.29) | 0.312 | 1.08 (0.91-1.28) | 0.395 | 1.11 (0.93-1.33) | 0.230 |
| Q4 | 1.22 (1.04-1.45) | 0.017* | 1.21 (1.02-1.43) | 0.026* | 1.31 (1.09-1.57) | 0.004* |

Model 1 = Crude

Model 2 = age, gender, race, education level, marital status, PIR, were adjusted

Model 3 = Model 2 + smoking status, drinking status, PA, diabetes, hypertension, and CVD, were adjusted

Abbreviations: OR, odds ratio; CI, confidence interval; HR, hazard ratio.

*P<0.05
