# Supplementary material for: Association of C‐Reactive Protein‐Triglyceride Glucose Index With Chronic Obstructive Pulmonary Disease: Results From the NHANES and CHARLS Cohorts
Source: Mediators Inflamm. 2026 Jul 4;2026:9592487. doi: 10.1155/mi/9592487 (PMC13332394; doi:10.1155/mi/9592487)
Supplement: Supplementary file 5 — Supporting Information 5 Table S5: Association between CTI and the risk of COPD after multiple imputation. [file MI-2026-9592487-s005.docx]

**Table S5** Association between CTI and the risk of COPD after multiple imputation.

| NHANES | Model 1 | | Model 2 | | Model 3 | |
| --- | --- | --- | --- | --- | --- | --- |
|  | OR(95%CI) | *P* Value | OR(95%CI) | *P* Value | OR(95%CI) | *P* Value |
| **CTI** | 1.57 (1.43-1.73) | <0.001* | 1.45 (1.31-1.60) | <0.001* | 1.30 (1.14-1.49) | <0.001* |
| **CTI Group** |  |  |  |  |  |  |
| Q1 | Ref. |  | Ref. |  | Ref. |  |
| Q2 | 1.85 (1.35-2.53) | <0.001* | 1.66 (1.19-2.32) | 0.003* | 1.49 (1.04-2.12) | 0.028* |
| Q3 | 1.85 (1.33-2.57) | <0.001* | 1.53 (1.09-2.16) | 0.014* | 1.26 (0.87-1.83) | 0.228 |
| Q4 | 3.24 (2.47-4.26) | <0.001* | 2.56 (1.95-3.37) | <0.001* | 1.93 (1.39-2.68) | <0.001* |
|  | | | | | | |
| CHARLS | Model 1 | | Model 2 | | Model 3 | |
|  | HR(95%CI) | *P* Value | HR(95%CI) | *P* Value | HR(95%CI) | *P* Value |
| **CTI** | 1.11 (1.04-1.19) | 0.002* | 1.11 (1.04-1.19) | 0.003* | 1.15 (1.06-1.24) | <0.001* |
| **CTI Group** |  |  |  |  |  |  |
| Q1 | Ref. |  | Ref. |  | Ref. |  |
| Q2 | 0.94 (0.79-1.12) | 0.469 | 0.91 (0.76-1.08) | 0.283 | 0.92 (0.77-1.10) | 0.367 |
| Q3 | 1.11 (0.93-1.31) | 0.242 | 1.09 (0.92-1.29) | 0.326 | 1.11 (0.93-1.32) | 0.241 |
| Q4 | 1.23 (1.04-1.45) | 0.016* | 1.21 (1.02-1.43) | 0.026* | 1.28 (1.07-1.53) | 0.008* |

Model 1 = Crude

Model 2 = age, gender, race, education level, marital status, PIR, were adjusted

Model 3 = Model 2 + smoking status, drinking status, PA, diabetes, hypertension, and CVD, were adjusted

Abbreviations: OR, odds ratio; CI, confidence interval; HR, hazard ratio.

*P<0.05
