# Supplementary material for: Association of C‐Reactive Protein‐Triglyceride Glucose Index With Chronic Obstructive Pulmonary Disease: Results From the NHANES and CHARLS Cohorts
Source: Mediators Inflamm. 2026 Jul 4;2026:9592487. doi: 10.1155/mi/9592487 (PMC13332394; doi:10.1155/mi/9592487)
Supplement: Supplementary file 6 — Supporting Information 6 Table S6: Association between CTI and the risk of COPD using unweighted logistic regression. [file MI-2026-9592487-s006.docx]

**Table S6** Association between CTI and the risk of COPD using unweighted logistic regression

| NHANES | Model 1 | | Model 2 | | Model 3 | |
| --- | --- | --- | --- | --- | --- | --- |
|  | OR(95%CI) | *P* Value | OR(95%CI) | *P* Value | OR(95%CI) | *P* Value |
| **CTI** | 1.46 (1.34-1.59) | <0.001* | 1.40 (1.28-1.53) | <0.001* | 1.28 (1.15-1.42) | <0.001* |
| **CTI Group** |  |  |  |  |  |  |
| Q1 | Ref. |  | Ref. |  | Ref. |  |
| Q2 | 1.79 (1.38-2.34) | <0.001* | 1.61 (1.23-2.11) | 0.001* | 1.49 (1.12-1.97) | 0.006* |
| Q3 | 1.85 (1.43-2.42) | <0.001* | 1.57 (1.20-2.07) | 0.001* | 1.37 (1.03-1.84) | 0.034* |
| Q4 | 2.85 (2.23-3.67) | <0.001* | 2.44 (1.90-3.17) | <0.001* | 1.92 (1.44-2.58) | <0.001* |

Model 1 = Crude

Model 2 = age, gender, race, education level, marital status, PIR, were adjusted

Model 3 = Model 2 + smoking status, drinking status, BMI, diabetes, hypertension, and CVD, were adjusted

Abbreviations: OR, odds ratio; CI, confidence interval.

*P<0.05
