# Supplementary material for: Association of C‐Reactive Protein‐Triglyceride Glucose Index With Chronic Obstructive Pulmonary Disease: Results From the NHANES and CHARLS Cohorts
Source: Mediators Inflamm. 2026 Jul 4;2026:9592487. doi: 10.1155/mi/9592487 (PMC13332394; doi:10.1155/mi/9592487)
Supplement: Supplementary file 7 — Supporting Information 7 Table S7: Association between CTI and the risk of COPD excluding participants with COPD during the first follow‐up period. [file MI-2026-9592487-s007.docx]

**Table S7** Association between CTI and the risk of COPD excluding participants with COPD during the first follow-up period.

| CHARLS | Model 1 | | Model 2 | | Model 3 | |
| --- | --- | --- | --- | --- | --- | --- |
|  | HR(95%CI) | *P* Value | HR(95%CI) | *P* Value | HR(95%CI) | *P* Value |
| **CTI** | 1.10 (1.02-1.18) | 0.010* | 1.10 (1.02-1.18) | 0.012* | 1.14 (1.05-1.24) | 0.002* |
| **CTI Group** |  |  |  |  |  |  |
| Q1 | Ref. |  | Ref. |  | Ref. |  |
| Q2 | 0.95 (0.79-1.14) | 0.601 | 0.92 (0.77-1.11) | 0.394 | 0.94 (0.78-1.13) | 0.501 |
| Q3 | 1.07 (0.90-1.27) | 0.468 | 1.05 (0.88-1.25) | 0.590 | 1.07 (0.90-1.29) | 0.447 |
| Q4 | 1.20 (1.01-1.42) | 0.044* | 1.18 (0.99-1.40) | 0.026* | 1.25 (1.03-1.51) | 0.021* |

Model 1 = Crude

Model 2 = age, gender, race, education level, marital status, PIR, were adjusted

Model 3 = Model 2 + smoking status, drinking status, BMI, diabetes, hypertension, and CVD, were adjusted

Abbreviations: OR, odds ratio; CI, confidence interval; HR, hazard ratio.

*P<0.05
